# Supplementary material for: Standardized surgical technique for single-port totally extraperitoneal inguinal hernia repair using the glove method with an intraumbilical incision
Source: PLoS One. 2025 Nov 4;20(11):e0334049. doi: 10.1371/journal.pone.0334049 (PMC12585077; doi:10.1371/journal.pone.0334049)
Supplement: Attachment — Submitted filename: Step hernia repair.pdf [file pone.0334049.s003.pdf]

Dear authors,

Thank you for the opportunity to review the paper titled “Standardized Surgical Technique for Single-Port Totally Extraperitoneal Inguinal Hernia Repair Using the Glove Method with an Intraumbilical Incision.”
Below are my comments

Abstract: line 40-42: we present the surgical outcomes at our institution for a surgeon in their eighth year of practice with 40 cases of experience with STEP in this surgical procedure.
Mentioning the volume of cases done before is confusing to the readers. Was the purpose of this to verify that surgeon was beyond the learning curve? If so, please clarify it in the methodology, and perhaps reflect on how that is relevant in general when learning new techniques in the discussion.

Line 99: We present a standardized STEP technique.. – Was this described before? Is it standardized internationally or locally in your service
This statement doesn’t state the aim of this paper clearly. To me this seems to be a paper assessing feasibility of a technique modification with a young surgeon, this involves ease of learning, and safety (patient related outcomes).

Choice of aim will reflect on outcomes. From surgeon’s perspective: discomfort/ease of operating, operative time, #cases during learning curve (reported later), need for conversion or redo
From a patient’s perspective, post-op pain, infection, need for admission, need for re-operation.
It would be helpful to provide a control group (if real-time is not available, then historical) for CTEP in your institution and how it compares to STEP.

Line 117: “From April to October 2023, he performed 29 STEP procedures with the same assistance” - Verify the total number of cases 25 vs 29

Choice of words: it might be better to use standard terminology such as early career surgeon instead of young surgeon. The latter reflects on age more than experience.
