# Supplementary material for: Standardized surgical technique for single-port totally extraperitoneal inguinal hernia repair using the glove method with an intraumbilical incision
Source: PLoS One. 2025 Nov 4;20(11):e0334049. doi: 10.1371/journal.pone.0334049 (PMC12585077; doi:10.1371/journal.pone.0334049)
Supplement: Attachment — Submitted filename: Response to Editor - 2.docx [file pone.0334049.s004.docx]

## **Title:** **Standardized Surgical Technique for Single-Port Totally Extraperitoneal Inguinal Hernia Repair Using the Glove Method with an Intraumbilical Incision**

Dear Paula Katrina A. Maderazo

Thank you for your guidance. We have carefully addressed all the issues you pointed out, and the revised manuscript has been updated accordingly. We hope the current version meets the requirements for further consideration.

**Comment 1**

*We note that there is identifying data in the* *Supporting Information file < raw data.xlsx >. Due to the inclusion of these potentially identifying data, we have removed this file from your file inventory. Prior to sharing human research participant data, authors should consult with an ethics committee to ensure data are shared in accordance with participant consent and all applicable local laws.*

**Response to comment 1**

**Thank you for your careful review and for pointing this out. We understand the importance of protecting participant privacy. In response, we have revised the Supporting Information file by removing the age data and any potentially identifying information. The revised, anonymized dataset has now been prepared and resubmitted as Supporting Information file < raw data new.xlsx >.**

**Comment 2**

*May you please confirm or clarify if Yoshiro Imai & Yoshiharu Miyamoto is just the same person?*

**Response to comment 2**

**Thank you for your inquiry. We confirm that Yoshiro Imai and Yoshiharu Miyamoto are not the same person.**

**Comment 3**

*We note that your author list was updated during the revision process. In order to add or remove authors or update the order of the author byline after initial submission, we ask that authors complete an Authorship Change Request form. You may review our full authorship change policy and download the Authorship Change Request form here:*

**Response to comment 3**

**Thank you for your message. We understand that any changes to the author list after the initial submission require completion of the Authorship Change Request form. We will complete the required form and submit it according to the journal’s policy.**
