# Supplementary material for: Standardized surgical technique for single-port totally extraperitoneal inguinal hernia repair using the glove method with an intraumbilical incision
Source: PLoS One. 2025 Nov 4;20(11):e0334049. doi: 10.1371/journal.pone.0334049 (PMC12585077; doi:10.1371/journal.pone.0334049)
Supplement: Attachment — Submitted filename: Response to Reviewers 3.docx [file pone.0334049.s005.docx]

## **Title:** **Standardized Surgical Technique for Single-Port Totally Extraperitoneal Inguinal Hernia Repair Using the Glove Method with an Intraumbilical Incision**

Dear Editor:

We thank you and the reviewers for your thoughtful suggestions and insights. The manuscript has benefited from these insightful suggestions. I look forward to working with you and the reviewers to move this manuscript closer to publication in *PLOSONE.*

The manuscript has been rechecked and the necessary changes have been made (using track changes mode/red font) in accordance with the reviewers’ suggestions. The responses to all comments have been prepared and attached herewith/given below. We have also had native English speakers check our manuscript.

Thank you for your consideration. I look forward to hearing from you.

**Responses to Reviewer Comments**

**To Reviewer**

**Reviewer’s comment**

*If the reviewer comments include a recommendation to cite specific previously published works, please review and evaluate these publications to determine whether they are relevant and should be cited. There is no requirement to cite these works unless the editor has indicated otherwise.*

**Response to comment**

**Thank you very much for your comment.**

**As no specific references were recommended by the reviewer, we did not make any additional citations.**
